# Hospital out-lying through lack of beds and its impact on care and patient outcome

**DOI:** 10.1186/1757-7241-21-17

**Published:** 2013-03-14

**Authors:** Andrew Stowell, Pierre-Geraud Claret, Mustapha Sebbane, Xavier Bobbia, Charlotte Boyard, Romain Genre Grandpierre, Alexandre Moreau, Jean-Emmanuel de La Coussaye

**Affiliations:** 1 Structure des urgences, CHU de Nîmes, place du Professeur Debré, Nîmes, 30029, France; 2 Structure des urgences, CHU de Montpellier, 191 Avenue du Doyen Giraud, Montpellier, 34295, France

**Keywords:** Emergency service, Hospital bed capacity, Patient admission, Hospitals, Organization

## Abstract

**Background:**

When medical wards become saturated, the common practice is to resort to outlying patients in another ward until a bed becomes free.

**Objectives:**

Compare the quality of care provided for inpatients who are outlying (O) in inappropriate wards because of lack of vacant beds in appropriate specialty wards to the care given to non outlying (NO) patients.

**Methods:**

We propose a matched-pair cluster study. The exposed group consisted of inpatients that were outliers in inappropriate wards because of lack of available beds. Non-exposed subjects (the control group) were those patients who were hospitalized in the ward that corresponded to the reason for their admission. Each patient of the exposed group was matched to a specific control subject. The principal objective was to prospectively measure differences in the length of hospital stays, the secondary objectives were to assess mortality, rate of re-admission at 28 days, and rate of transfer into intensive care.

**Results:**

238 were included in the NO group, 245 in the O group. More patients in the O group (86% vs 76%) were transferred into a ward with prescription completed. O patients remained in hospital for 8 days [4-15] vs 7 days [4-13] for NO patients (p = 0.04). 124 (52%) of the NO patients received heparin-based thromboembolic prevention during their stay in hospital vs 104 (42%) of the O patient group (p = 0.03). 66 (27%) O patients were re-admitted to hospital within 28 days vs 40 (17%) NO patients (p = 0.008).

**Conclusion:**

O patients had a worse prognosis than NO patients.

## Introduction

French hospitals and their emergency departments (ED) are becoming more and more saturated. Throughout the world, studies have shown a link between overcrowding in an ED and a lowering of treatment provided in that ED [1,2]. It has been shown that hospitalization in an overcrowded ED results in increased mortality at 10 days [3], decreased quality of pain care [4], higher incidence of re-admission and repeated visits to the emergency room [1,2]. The lack of beds downstream brings about longer boarding times in the ED before hospital admission. This has been described in a number of countries, including Australia [5], the U.K. [5,6] and North America [7] as one of the principal causes of overcrowding in the triage areas. More than half of the patients (57.7%) say that this lack of beds caused too long a wait in the ED [8]. In pediatrics, the overly-high bed occupancy rate in the ward is seen as a factor in longer ED boarding times [9]. Boarding in the ED has a negative effect on patient care [10] and increases the average length of hospital stay [11,12]. Reducing admission time would help bring down rates of mortality and admissions later to intensive care [13]. This is especially true for critically-ill patients [14].

When particular wards are saturated, the common practice is to resort to outlying patients in another ward until a bed becomes free in the adequate ward [15]. The outlying patients therefore receive medical care from one ward but remain under the responsibility of doctors in the appropriate wards. Such temporary outlying may sometimes extend to the whole period of hospitalization.

Few studies have been undertaken regarding the practice of outlying patients [16-19]. Yet the presence of outlaid patients poses basic questions about the quality of care provided to those patients. Surgical patients may be outlying in medical wards and vice versa. Is the care provided the same as they would have received if they had been admitted straight into their destination ward? Do staff provide adequate care to outlying patients? In the U.K., it has been shown that there is a lack of knowledge among nurses in medical wards about caring for surgical patients compared to nurses in orthopedic or traumatology wards [16,17]. A study in France has emphasized the need for designating a contact doctor in wards that have outlying patients in other wards, as well as a nursing care coordinator and making sure medical files are standardized throughout the hospital [18]. Previous studies only looked at medical care delivered by paramedics on surgical or traumatology patients and did not give any description of the outcomes of those patients [16,17].

The aim of our study was to compare the care afforded to outlying patients with the care given to non outlying patients by analyzing their outcomes at 3 months after hospitalization. Our objective was to evaluate the practice of outlying and its impact on patient care using a set of criteria that enabled us to assess the quality of care.

## Methods

### Study objectives

The principal objective was to measure differences in length of hospital stays between outlying inpatients and non outlying inpatients, i.e. those directed straight to their destination ward.

The secondary objectives were to assess mortality at 24 hours, 28 days and 3 months; the rate of readmission to hospital at 28 days; the rate of transfer into the intensive care unit (ICU) and to identify the reasons why hospitalization in outlying patients is less efficient.

### Type of study

The study took place from February to June 2010 in the ED of a university hospital with an annual census of 60 000 patients in a major town in the south of France with 150 000 citizens. This was a monocentric prospective study of matched-pair clusters in an exposed/non-exposed cohort. In the exposed group we examined those patients who had been hospitalized in a medical or surgical ward as outliers, while the non-exposed subjects were those patients hospitalized in their destination ward. Clusters were paired on the basis of age, sex and type of pathology that had caused hospitalization. The anticipated duration of patient participation was 90 days, i.e. the time needed to establish the rate of mortality at 90 days. According to French law (Law 88–1138 of the 20th of December 1988 relative to Biomedical Research as amended on the 9th of August 2004), this non-interventional study did not require approval by an ethics committee nor informed signed consent from patients. It was reviewed and approved by our institutional review board. Moreover, the present study was declared to the national commission for data processing and civil liberties (the CNIL) and approved by them (declaration number 1513822).

All data were kept confidential and used only for research. The electronic data were stored in a password-protected computer. The investigators had complete independence in developing the survey, collecting the data, analyzing the data and reporting the results.

### Selection and inclusion of patients

Patients were selected from a period running from February to May 2010. Criteria for inclusion were any patient outlying in one ward but under the responsibility of another ward. Surgical wards were considered to be orthopedics, otolaryngology, thoracic and vascular surgery, urology and gynecology. Medical wards were considered to be internal medicine, infectious, oncology, hematology, pneumology, geriatric medicine, nephrology and cardiology. Criteria for non-inclusion were: refusal to take part in the study, persons under judicial protection or guardianship, persons under 18 years old, and patients hospitalised directly in intensive care units from the ED. Pregnancy was not considered to be a criterion for non-inclusion.

### Data collection

The first group included the outlying patients after initial randomisation. The second group was the control group including the non out-lying patients (for example, patient with pneumonia hospitalized in the respiratory ward). Patients of this second group were consecutively included among all patients hospitalized during the study period. They were matched with the first group according to age (within five years), sex and reason for admission (within pathological groups: cardiovascular, gastroenterology, neurology, pneumology and trauma). Each patient of the first group (out-lying patients) was matched to a specific control patient in the second group (non out-lying patients).

We noted the care provided to patients from their arrival in casualty until they were released from hospital and their outcome up to 90 days after hospitalization. Data collection was therefore carried out in two phases. In the first phase, from March to May 2010, we were able to collect information concerning the hospitalization of each patient using the patient care tracking software (Clinicom, Siemens Health Services, Munich, Germany). Medical files gave access to administrative data (date and time of arrival in the ED, arrival in triage and subsequent movements and the various wards in which the patient was hospitalized), clinical and para-clinical data (biological and imagery tests done during hospitalization), the name of the doctor who provided emergency care (senior or junior) and computerized prescription with the name of the prescribing physician (senior or junior) and the date of each prescription. In the second phase, patients included in the study (or their family) were contacted by phone in July and August 2010 and questioned as to mortality at 28 days and 90 days and as to any re-admission to hospital at 28 days. The starting point for these durations was taken as the date of arrival in the ED.

### Outcome criteria

The main outcome criterion was the duration of stay in hospital (day, mean, SD). Secondary criteria were mortality rates at 24 hours, 28 days and 90 days from admission to ED; transfer to intensive care; re-admission to hospital at 28 days from admission to ED and prevention of thromboembolism with low molecular weight heparin (hospital protocol suggests systematic use of trombo-embolic stockings with, prescription of a low mechanical weight heparin is left to the practitioner).

### Statistics

The length of stay in our hospital ranged from 0 to 369 days, i.e. an average duration of 7.56 +/− 12.1. This estimate is an over-estimate due to long-term hospitalization, with a median of 3.5 days. When we concentrated on 95% of short hospital stays, i.e. between 0 and 28 days, the average duration was 5.5 +/− 5.86 days. This was the estimate used in deciding the number of subjects. The main study assumption was to consider an increase in the duration of the stay in hospital due to outlying that was significant and clinically relevant. In principal, this corresponded to an increase of at least 25% of the average hospital stay. An initial processing of data for the patients in the exposed and the non-exposed groups produced qualitative variables (using frequencies and percentages) and quantitative variables (using means and standard deviation or median with inter-quartile depending on type of distribution). A Student test was used to establish and compare the main outcome criterion (length of hospital stay) for the two groups of patients. Student, the chi-square, Fisher exact test and Mann and Whitney tests were used where appropriate in order to compare the parameters studied in exposed and non-exposed populations. Data was analyzed and represented with R project (free software foundation, GNU general public license), Numbers (Apple, Cupertino, CA) and Excel (Microsoft, Santa Rosa, CA). In all analyses, two-sided P values of less than 0.05 were considered to indicate statistical significance.

## Results

### Baseline characteristics

**Figure 1:**
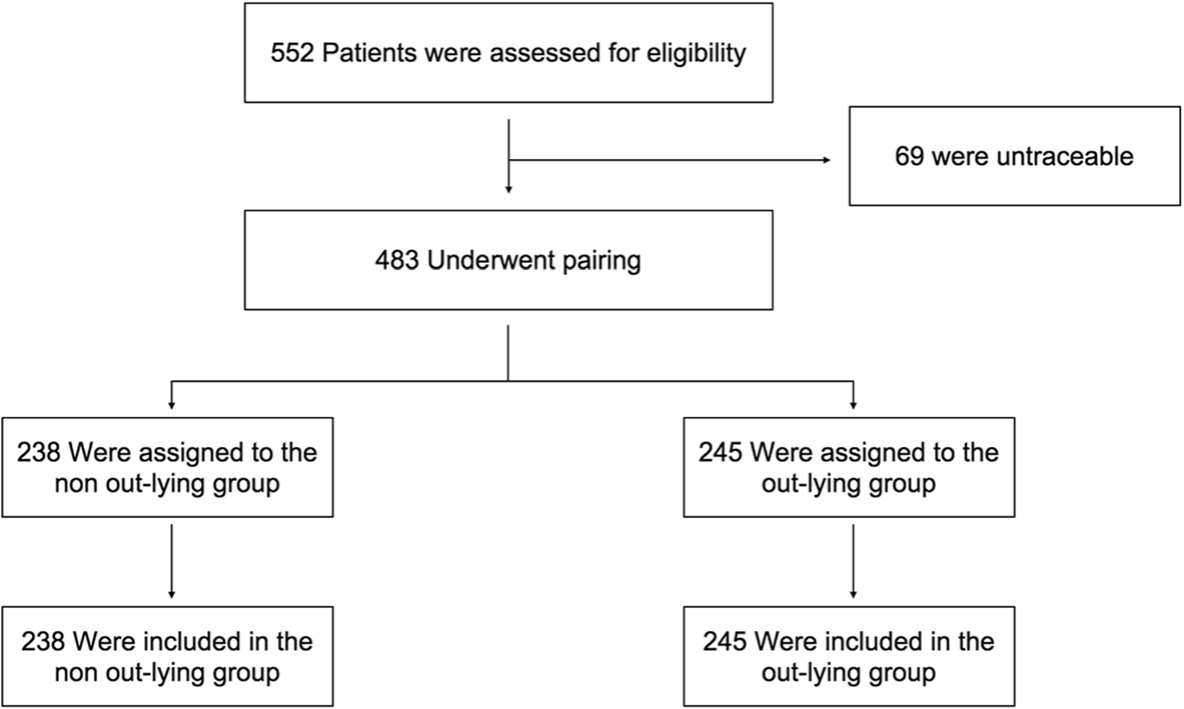
Flowchart.

552 patients were initially included, of which 69 were untraceable. 238 patients were included in the non outlying group, 245 patients were included in the outlying group (Figure 1). Age, sex ratio, reasons for admission and biological characteristics of the two groups did not present any significant differences (Table 1).

**Table 1:** Baseline characteristics

	**Non out-lying**	**Out-lying**
Age – median year (IC)	76 (58 – 85)	69 (54 – 80)
Male – no. (%)	119 (50%)	122 (49.79)
Reason for admission:		
Cardiovascular – no. (%)	35 (14.71)	32 (13.06)
Gastroenterology – no. (%)	39 (16.39)	42 (17.14)
Neurology – no. (%)	80 (33.61)	79 (32.24)
Pneumology – no. (%)	60 (25.21)	65 (26.53)
Trauma – no. (%)	28 (11.76)	39 (15.92)
Haemoglobine – mean g/dL (SD)	13.03 (2.22)	12.80 (2.17)
White blood cells – mean G/L (SD)	10.67 (5.01)	10.78 (8.36)
Creatinine – mean μmol/L (SD)	92.60 (57.75)	93.76 (72.69)

### Care in the emergency department

More patients in the outlying group (211 [86%] vs 181 [76%], p = 0.005) were transferred into a ward with prescription completed. Hospital policy is to leave the prescription to the clinician accepting the patient in his ward except at night. We also found a tendency for a higher number of LMWH (low molecular weight heparin) prescriptions (74 [31%] vs 58 [24%], p = 0.08) in the non outlying patient group. Data relating to other ED care are set out- in Table 2.

**Table 2:** Care in emergency department

	**Non out-lying**	**Out-lying**
Admitted to resuscitation room – no. (%)	65 (27.31)	76 (31.02)
First examination by resident/medical student – no. (%)	102 (42.86)	109 (44.49)
ED length of stay – median hour (25% – 75%)	10 (6 – 16)	9 (6 – 14)
Prescriptions written in ED for wards – no. (%)	181 (76.05)	211 (86.12) **
LMWH prescribed by ED – no. (%)	74 (31.09)	58 (23.67)

p-value < 0.05.

** p-value < 0.01.

*LMWH*: Low Molecular Weight Heparin.

### Care in wards

Outlying patients remained in the hospital for 8 days [4-15] compared to 7 days [4-13] for non-outlying patients (p = 0.04). 124 (52%) of the non-outlying patients received heparin-based thromboembolic prevention during their stay in hospital compared to 104 (42%) of the outlying patient group (p = 0.03). Data relating to other wards care are set out in Table 3.

**Table 3:** Care in wards

	**Non out-lying**	**Out-lying**
Wards length of stay – median day (IC)	7 (4 – 13)	8 (4 – 15) *
Length of stay as out-lying – median day (IC)		1 (1 – 3)
LMWH prescribed during hospitalization – no. (%)	124 (52.10)	104 (42.45) *
Number of biological test during hospitalization (SD)	4.59 (4.57)	5.13 (7.32)
Number of imagery test during hospitalization (SD)	1.41 (2.07)	1.65 (2.47)

* p-value < 0.05.

p-value < 0.01.

*LMWH*: Low Molecular Weight Heparin.

### Clinical outcomes

66 (26%) outlying patients were readmitted to hospital within 28 days compared to 40 (17%) non outlying patients (p = 0.008). Data relating to mortality and transfers into intensive care unit are set out in Table 4.

**Table 4:** Clinical outcomes

	**Non out-lying**	**Out-lying**
Transferred to intensive care unit – no. (%)	13 (5.46)	14 (5.71)
Re-admitted in hospital within 28 days – no. (%)	40 (16.81)	66 (26.94) **
Death within 24 hours – no. (%)	2 (0.84)	0 (0.00)
Death within 28 days – no. (%)	33 (13.87)	25 (10.20)
Death within 90 days – no. (%)	49 (20.59)	38 (15.51)

p-value < 0.05.

** p-value < 0.01.

## Discussion

552 patients were included, 238 in the non outlying group, 245 in the outlying group. In the outlying group, more patients were transferred into a ward with prescription completed. In this group, patients received less heparin-based thromboembolic prevention during their stay in hospital and remained in hospital longer. Moreover, outlying patients were more often readmitted to hospital within 28 days compared to non outlying patients.

Few studies have been undertaken of outlying patients [16-18]. There have been some studies highlighting the risk exposure of outlying patients but these only took into account paramedical care and only studied patients in surgery and traumatology [16,17]. A Spanish study did conclude to longer length of stay for outliers with heart failure [19]. However, we have not identified any studies having looked at the outcomes of outlying patients within several diagnosis groups. The outlying group and the non outlying group had the same demographic characteristics and had the same degree of seriousness as demonstrated by their biological data and the proportion of them receiving care in the resuscitation room. We found the same care in the ED as demonstrated by the proportion of patients initially receiving care from senior doctors. This should be measured against the work of Alison L. White in the U.K. who found lower standards of care when patients were seen by junior doctors [19]. The duration of stay in the ED was not statistically different. This result would tend to refute the preconceived idea that outlier patients stay longer in the ED i.e. the time needed to find them a bed. This is even more interesting since the length of stay in the ED has been found to be a factor in the risk of increasing the average stay in hospital [11,12]. According to Gilligan et al., the length of stay in the overcrowded ED in the U.K. is a factor in the risk of mortality and also in contracting Methicillin-resistant Staphylococcus aurous infections.

### Outlying: hospitalization with greater risk

The average hospital stay is an important piece of data that is often correlated with complications. In our study we observed a significant increase in the average hospital stay among outlying patients. The reasons for this are certainly numerous and varied and our study was not sufficient to identify them clearly. We can, therefore, only put forward a number of possibilities including the following: longer delay between the time of arrival in the outlying ward and first contact with a medical practitioner; insufficient medical contact between the outlying patient and the doctors in the outlying ward; a standard of care that is not as good as a specialist ward would provide; a lack of knowledge among medical nurses when caring for surgical or traumatology patients [16,18].

Whatever assumptions are finally accepted, it remains true that outlying patients have identified as being a population at risk of developing complications because they stay in hospital longer.

### Greater number of re-admissions among outlying patients

The results of our study show a larger number of readmissions at 28 days in outlying patients. This finding enables us to affirm that outlying is a factor in the risk of early patient readmission. The risk factors on readmission are numerous. In the literature we find multiple co-morbidity [20], functional impotence [21,22], age [20,21], multiple recent stays in hospital [20], low social level [23,24] and a history of depression [20].

The French study by Lepage B et al. [18] brought out a number of mistakes made when discharging outlying patients, which could go some way toward explaining our results. We found several of these mistakes to be significant, particularly the medical advice given to patients on the day they are discharged (including the discharge papers and prescriptions). The advice and papers are not necessarily dealt with by a specialist doctor from the ward in charge of the outlying patient. In addition the follow-up appointment is not systematically organized by the specialist doctor.

### Thromboembolic prevention neglected in outlying patients

We observed that in an ED there is a tendency to prescribe more LMWH to non outlying patients than to outlying patients. One of the reasons could be concern regarding the side effects of heparin treatment due to the less effective medical monitoring in the outlying ward. Our results show that non outlying patients benefited in their ward more often from anti-thrombosis prophylactic treatment with heparin and this confirms the tendency observed with ED prescriptions. In the USA thromboembolic prevention has been singled out as one of the most important quality criteria of care for inpatients [25,26]. Thromboembolic complications are the number one cause of avoidable mortality during a stay in hospital [25]. The ENDORSE study [27], which set out to study the prevalence of thromboembolic complications in hospital inpatients, found that of the 68 183 patients included (64.4% of surgical patients and 41.5% of medical patients showed a risk of complications), only 58.5% of those patients had benefited from prophylactic measures. If we take the ENDORSE study [27] as a basis then outlying patients with the risk of thromboembolic complications are even more at risk because they are outliers.

### Biology and imagery tests in the ward

Even though the average length of stay in hospital is significantly greater in outlying patients, we were not able to find any evidence of any difference in absolute terms in the number of biology or imagery tests. However, since their hospital stay lasts longer, the ratio of tests over length of stay is greater in non outlying patients than in outlying patients.

### More prescriptions for outlying patients in ED

The percentage of prescriptions written in an ED for patients hospitalized as outliers is greater than for patients from the non outlying group. The reasons for this could be: possible excess medication upstream due to in situ or telephoned advice received by emergency staff; concern in the outlying ward that they are not giving enough medication.

### Limits of the study

The study was performed at a single institution and our results may not be representative of other institutions. Pairing of patients was done based on age, sex and reasons for hospitalization. It would have been interesting to pair them by hospital ward and also using a severity injury score so as to obtain a better comparison of the two groups. Certain data relating to the lapse of time between arrival in the ward and first contact with a doctor would be interesting to analyze so as to explain the difference in average hospital length of stay.

We also may have found other significant differences if we had exclusively studied a population of medical patients outlying in surgical wards. In our study 80% of the patients were medical patients outlying in other medical wards.

Quality of care was mainly evaluated using the average length of stay. Considering the numerous wards included in our study other criterias could have been used.

At last, the study does not take into account many variables such as hospital occupancy or even ratios between the number of patients and nurses or between patients and doctors that differ from one ward to another.

## Conclusions

We set out to evaluate the quality of care provided for inpatients of a teaching hospital outlying in inappropriate wards because of lack of vacant beds in appropriate specialty wards and the impact on their outcomes by conducting a monocentric prospective study of a cohort. Our study has its limits, but the sample studied remains representative. It is important to bear in mind that outlying, although not the optimal solution, is most likely better than having an increased number of boarding patients in the ED. To our knowledge, it is the only study that enables a significant comparison of the care received by outlying patients and that received by non outliers. Our results enabled us to conclude that outlying patients receive a lower standard of care and this exposes the patients to an increase in the average length of their hospital stay, early readmission to the hospital and insufficient thromboembolic prevention. It therefore cannot be denied that the practice of outlying is at risk. It is important that emergency doctors and hospital mangers should be informed of risks inherent to outlying a patient due to lack of space in the right ward.

## Competing interests

The authors have no commercial associations or sources of support that might pose a conflict of interest.

## Authors’ contributions

PGC conceived the study and designed the trial. PGC and AS supervised the conduct of the trial and data collection. AS managed the data, including quality control. PGC provided statistical advice on study design and analyzed the data. PGC and AS drafted the manuscript, and all authors contributed substantially to its revision. PGC takes responsibility for the paper as a whole. All authors read and approved the final manuscript.
